# Potassium Current Is Not Affected by Long-Term Exposure to Ghrelin or GHRP-6 in Somatotropes GC Cells

**DOI:** 10.1155/2013/913792

**Published:** 2013-02-24

**Authors:** Belisario Domínguez Mancera, Eduardo Monjaraz Guzman, Jorge L. V. Flores-Hernández, Manuel Barrientos Morales, José M. Martínez Hernandez, Antonio Hernández Beltran, Patricia Cervantes Acosta

**Affiliations:** ^1^Laboratorio de Neuroendocrinología, Instituto de Fisiología, Benemérita Universidad Autónoma de Puebla, CP 7200, PUE, Mexico; ^2^Laboratorio de Biología Celular, Facultad de Medicina Veterinaria y Zootecnia, Universidad Veracruzana, CP 91710, VER, Mexico

## Abstract

Ghrelin is a growth hormone (GH) secretagogue (GHS) and GHRP-6 is a synthetic peptide analogue; both act through the GHS receptor. GH secretion depends directly on the intracellular concentration of Ca^2+^; this is determined from the intracellular reserves and by the entrance of Ca^2+^ through the voltage-dependent calcium channels, which are activated by the membrane depolarization. Membrane potential is mainly determined by K^+^ channels. In the present work, we investigated the effect of ghrelin (10 nM) or GHRP-6 (100 nM) for 96 h on functional expression of voltage-dependent K^+^ channels in rat somatotropes: GC cell line. Physiological patch-clamp whole-cell recording was used to register the K^+^ currents. With Cd^2+^ (1 mM) and tetrodotoxin (1 **μ**m) in the bath solution recording, three types of currents were characterized on the basis of their biophysical and pharmacological properties. GC cells showed a K^+^ current with a transitory component (*I*_A_) sensitive to 4-aminopyridine, which represents ~40% of the total outgoing current; a sustained component named delayed rectifier (*I*_K_), sensitive to tetraethylammonium; and a third type of K^+^ current was recorded at potentials more negative than −80 mV, permitting the entrance of K^+^ named inward rectifier (K_IR_). Chronic treatment with ghrelin or GHRP-6 did not modify the functional expression of K^+^ channels, without significant changes (*P* < 0.05) in the amplitudes of the three currents observed; in addition, there were no modifications in their biophysical properties and kinetic activation or inactivation.

## 1. Introduction

The growth hormone is mainly under the control of two hypothalamic neuropeptides acting in opposition: one, the growth hormone releasing hormone (GHRH), as a stimulant, and the other, somatostatin, as an inhibitor [1, 2]. The GHRH specifically bind to its receptor on the plasmatic membrane of the somatotropes; this increments the activity of adenylate cyclase, which increases the generation of AMPc [3, 4]. This increase in the AMPc levels let to open the voltage-dependent Ca^2+^ channels [5, 6] and a rapid increase in the intracellular Ca^2+^ concentration [Ca^2+^], thus promoting the exocytosis of GH [7, 8]. The inhibitory effect of somatostatin involves the inhibition of adenylate cyclase activity and a reduction of [Ca^2+^]_*i*_ [9, 10].

In addition to the GHRH, a group of synthetic oligopeptides releasing GH (GHRPs) or GH secretagogues (GHS) are capable of stimulating the secretion of GH [2, 11, 12]. The GH-releasing peptide-6 (GHRP-6) is one of the most representative of those compounds [2, 11–13]. Research on the mechanism of GHS action upon the liberation of GH led to the discovery of the GHS receptor, the GHS-R, and later to the ghrelin, the endogenous ligand for the GHS-R [2, 11, 12, 14].

It has been suggested that diverse forms of signaling are activated by the action of GHS. After binding the ligand, the GHS-R receptor acts through the subunit of the G protein to activate phospholipase C (PLC), which results in the hydrolysis of PlP_2_ to produce inositol 1,4,5-triphosphate (IP_3_) and diacylglycerol (DAG) [2]. As a consequence, there is an increase of [Ca^2+^]_*i*_ due to a transitory liberation of Ca^2+^ from the cytoplasmatic reserves sensitive to IP_3_ and to the sustained influx of Ca^2+^ caused by the activation of the voltage-dependent Ca^2+^ channels this in addition to the blockage of the K^+^ channels [16]; leads to a depolarization of the somatotrope membrane and the liberation of GH [2, 11]. The long-term effects of secretagogues like ghrelin and GHRPs on the cells secreting GH in association with the ionic channels explored have been few.

Cellular excitability depends on the opening and the closing of the ionic channels present in the plasmatic membrane as well as the level of membrane potential in repose. The conductance of K^+^ is responsible for the membrane potential in repose [17]. It has been reported that somatostatin can increase different types of K^+^ currents, including the voltage-gated K^+^ currents in somatotropes from rats, ovines, and humans in order to hyperpolarize the membrane potential [18, 19]. On the other hand, it has been reported that synthetic secretagogues reduce the KIR current (potassium inward rectifier) [15] by means of a decrease in the protein that codifies for K^+^; similarly, ghrelin has been reported to reduce the voltage-dependent K^+^ in GH_3_ cells via the GMP cycle [20]. Since most of the ionic currents passing through the plasmatic membrane in the membrane potential in repose are conducted by K^+^, it is believed that the K^+^ channels play an important role in the depolarization and repolarization induced by ghrelin [21]. Therefore, the object of the following was to examine whether chronic treatment with ghrelin or GHRP-6 modifies the functional expression of voltage-dependent K^+^ channels in the plasmatic membrane of GC cells during long-term treatments.

## 2. Material and Methods

### 2.1. Chemicals

Ghrelin (Cat. 55-0-03A) and GHRP-6 (Cat. 52-1-80B) were acquired from the American Peptide Company, Inc. (Sunnyvale, CA,USA). Tetrodotoxin (TTX; Cat. T550) was acquired from Almone Labs, Ltd. (Jerusalem, Israel). The chloride of tetraethylammonium (TEA; T2265) and the 4-aminopyridine (4-AP; A 0152) came from SIGMA (St. Louis, MO, USA); all other reagents were of a chemical grade.

### 2.2. Cell Culture

The cellular line of rat GC somatotropes was routinary maintained as a monolayer previously described [21] in a complete MegaCell DMEM culture medium (Sigma-Aldrich, St. Louis, MO, USA), supplemented with 3% of fetal bovine serum (Sigma-Aldrich) and 100 u.i. mL^−1^ of penicillin and 100 *μ*g mL^−1^ of streptomycin (Sigma-Aldrich). The cultures were incubated at 37°C in a humidified atmosphere containing 5%  CO_2_. Once a week the cells were harvested by means of a Trypsin-EDTA treatment (Sigma-Aldrich) (0.05 w/v and 453 mM, resp.) and reseeded at densities of 2–2.5 × 10^5^ cells per flask of 25 cm^2^. For electrophysiological recordings, the cells were seeded in culture dishes with poly-L-lysine for better cellular adhesion. The culture medium (with or without the secretagogues ghrelin or GHRP-6) was replaced every day.

### 2.3. Electrophysiology

The K^+^ currents were recorded in the cellular line of rat GC somatotropes under control conditions or after a chronic treatment (96 h) with ghrelin (10 nM) or GHRP-6 (100 nM) in the whole-cell recording configuration (WCR) of the patch-clamp technique [22]. For this, a patch-clamp amplifier Axopath 200B (Molecular Devices, Foster City, CA, USA) was used, as well as an interface Digidata 1322A with pClamp9 software (Molecular Devices) for the acquisition of on-line data. After the whole cell configuration was established, the capacitive transients were cancelled with the amplifier. The leakage current and the residual capacitive were subtracted online with a P/4 protocol. The current signals were filtered at 5 kHz (internal 4-pole Bessel filter) and digitalized at 10–100 kHz. The bath solution for recording contained (in mM): 145 NaCl,  5 KCl, 5 CaCl_2_, 10 Hepes, 1 CdCl_2_, 0.001 TTX, and 5 glucose; (pH 7.30, adjusted with NaOH). The internal solution for recording consisted of (in mM) 100 KCl, 30 NaCl, 2 MgCl_2_, 1 CaCl_2_, 10 EGTA, 10 Hepes, 2 ATP, 0.05 GTP, and 5 glucose (pH 7.30 adjusted with KOH). The experiments were performed at room temperature (~22°C). Both the control cells and the treated ones were rinsed in a culture medium free of peptides and were kept for ~60 min before the membrane currents were recorded in order to avoid acute effects. The membrane capacitance (Cm) was determined as previously described [23] and was used to obtain the current density. Briefly, the Cm values were determined by applying a series of consecutive pulses (20 ms; −20 mV) having a maintenance potential (−80 mV), and by integrating the trace of the current obtained by subtracting the capacitive transient traces associated with the pipette patch (cell-attached conditions) from the total of the capacitive transients obtained immediately after the interior of the cell was broken (whole-cell conditions).

### 2.4. Hormonal Quantification

The release of GH from GC cells treated with ghrelin (10 nM) or GHRP-6 (100 nM) for 96 h was quantified using a commercial enzyme-linked immunosorbent assay (ELISA) kit (SPI Bio, Massy Cedex, France) as previously described [21]. The color intensity of the reaction product (proportional to the concentration of GH) was measured by spectrophotometry in a plate reader using a 450 nm filter (Stat Fax 2100; Awareness Technology, Palm City, FL,USA). Intensity values of the samples were compared with values in a standard curve using SigmaPlot 11.0 software (Systat Software, Chicago, IL, USA).

### 2.5. Data Analysis

The data were analyzed and graphed by combining pClamp software (previously mentioned) and SigmaPlot software v.11 (SPSS, Chicago, IL, USA); they are shown as the mean ± standard error. The statistical differences between the means were determined with the Student *t*-test (*P* < 0.05). The adjustments to the curves were made by using nonlinear procedures by least squared included in the SigmaPlot program. The conductance of each testing potential was estimated by measuring the amplitude of the current at its peak. The conductance-voltage curves (*G*-*V*) for analyzing the activation were adjusted by means of a Boltzmann-type equation in the form of *G* = *G*_max_/(1 + exp[(*V*_*m*_ − *V*_1/2_)/*k*]^−1^), where *G*_max_ is the maximum of conductance, *V*_*m*_ is the testing potential, *V*_1/2_ is the potential for the half of *G*_max_ (midpoint), and *k* is a slope factor. To construct the inactivation curve a protocol of two pulses was used; the first of these was denominated as conditioning, with duration of 1.5 s and variable amplitude (−130 to 60 mV), and the second pulse as testing, the latter having a membrane potential at 70 mV with a duration of 500 ms; the amplitude was graphed at the peak of the type *I*_A_ current induced by the testing pulse, dependent on the voltage of the conditioning pulse. Subsequently, the data obtained from each cell were adjusted with a Boltzmann-type equation in the form of *I* = *I*_max_/(1 + exp[(*V*_*m*_ − *V*_1/2_)/*k*]^−1^), where *I*_max_ is the maximum current, *V*_*m*_ is the testing potential, *V*_1/2_ is the potential for the half of *I*_max_, and *k* is a slope factor.

## 3. Results

### 3.1. General Properties of the K^+^ Current in the Cellular Line of Rat GC Somatotropes under Control Conditions and Treated with Secretagogues (Ghrelin or GHRP-6)

**Figure 1:**
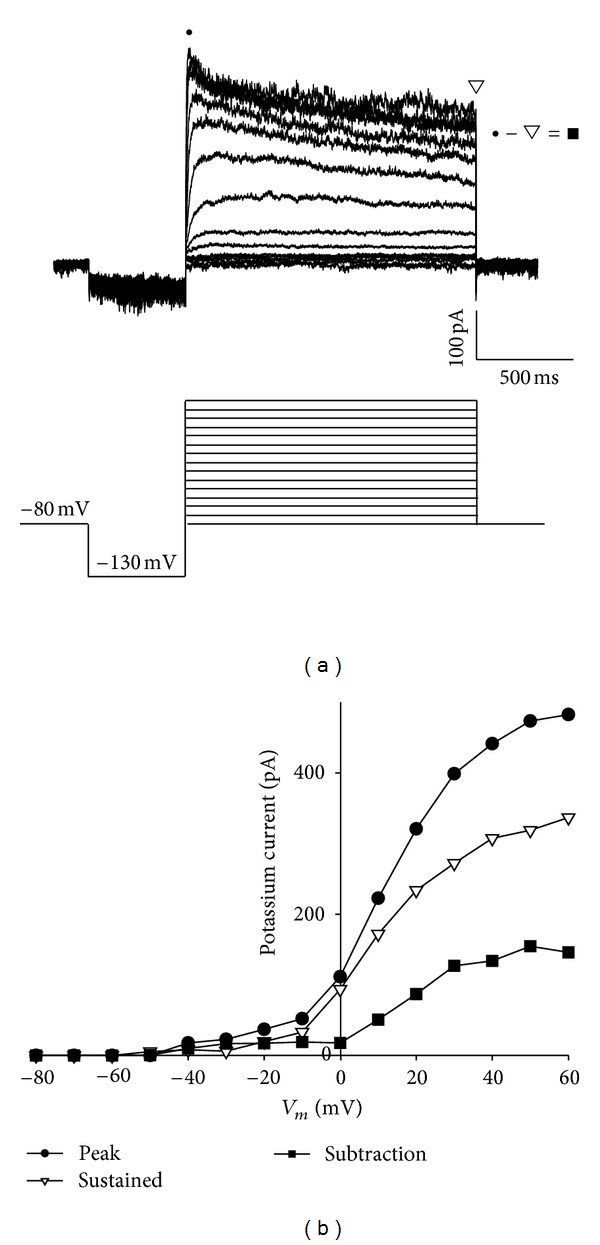
Voltage-dependent potassium current in the cellular line of rat GC somatotrope. (a) Family of representative traces of voltage-dependent K^+^ current in GC cells, evoked by depolarizing voltage pulses with increases of 10 mV, starting from a holding potential in the prepulse of −130 mV with duration of 500 ms, followed by a voltage pulse lasting for 1.5 sec. The initial maintenance potential was fixed at −80 mV. In the lower part, the acquisition protocol is shown. (b) Current-voltage relationship for the transient component (●; the first 50 ms), the sustained component (□; 1.455–1.50 s), and the subtraction (● − □ = ■).

The total current of voltage-dependent K^+^ in the GC cells was examined through the application of a pulse protocol, starting with a prepulse (duration of 500 ms) that fixes the holding potential at −130 mV; there followed a series of voltage pulses with a duration of 1.5 seconds, starting from −80 mV to 60 mV with increases of 10 mV (Figure 1(a), lower panel). In the GC cells, two components were observed in the potassium current, one transient component in the first 50 ms, and a sustained component that is maintained during the 1.5 seconds of the depolarizing pulse (Figure 1(a)); both the peak and the sustained part were activated at membrane potentials more depolarizing than −30 mV (Figure 1(b)). Once activated, the total current of voltage-dependent potassium inactivates slowly during the 1.5-second passing of depolarizing voltage (Figure 1(a)).

**Figure 2:**
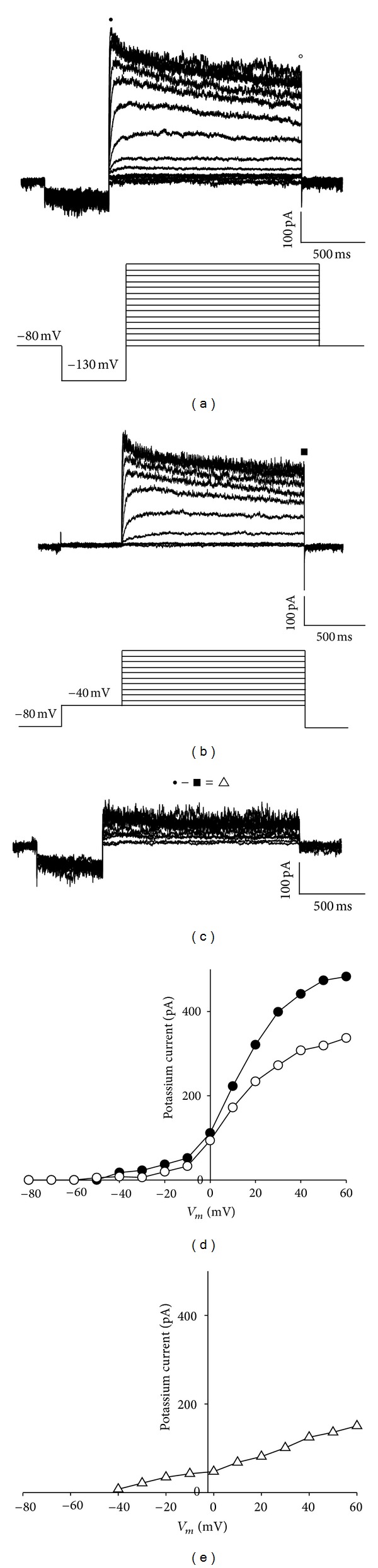
Components of the voltage-dependent potassium current in the cellular line of rat GC somatotropes. (a) Family of representative traces of the voltage-dependent K^+^ current in the GC cells evoked by depolarizing voltage pulses lasting 1.5 sec, with increases of 10 mV starting from a prepulse of 500 ms and a holding potential of −130 mV, the initial holding potential have been fixed at −80 mV. The lower part shows the acquisition protocol. (b) Representative traces of the voltage-dependent K^+^ current evoked by 1.5 s depolarizing pulses, starting from a prepulse lasting 500 ms and a holding potential of −40 mV, increasing by 10 mV at a time to 60 mV; the pulse protocol is shown in the lower part. (c) Transient K^+^ current (*I*_A_) isolated through a point-by-point subtraction of currents from traces (a) and (b): (● − ■ = □). (d) Current-voltage relationship at peak of current; transient component (●; the first 50 ms), and sustained component (□; 1.455–1.5 s). (e) Current-voltage relationship of the point-by-point subtraction between (a) and (b): the measurement was taken in the first 50 ms (● − ■ = □).

In order to isolate the slow-inactivation potassium current (*I*_K_) component, the transient potassium current (*I*_A_) was eliminated through a maneuver to change the holding potential from −130 mV to −40 mV for 500 ms in the prepulse, prior to the depolarizing pulses of 1.5 seconds ranging from −40 to 60 mV (Figure 2(b)). Subsequently, the point-by-point subtraction in the two families of current traces was performed for each potential, thereby obtaining the current of rapid activation and rapid inactivation known as *I*_A_. 

**Figure 3:**
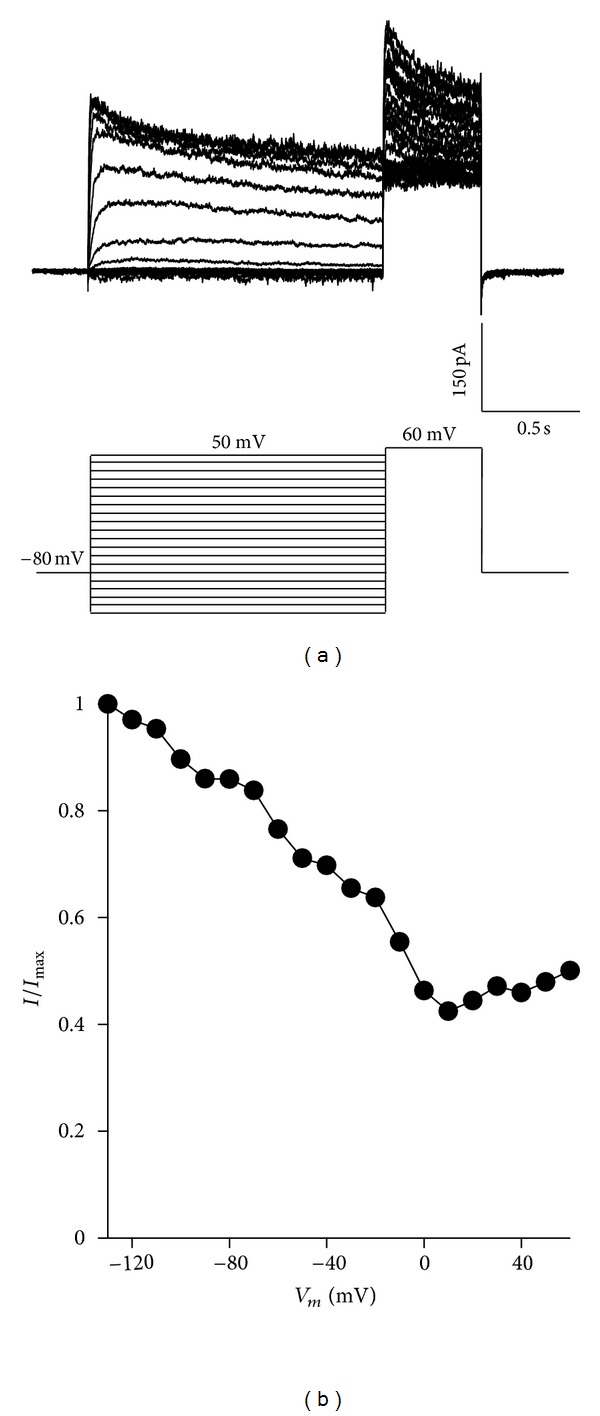
Inactivation in the steady state of the total *I*_K_ current in the cellular line of rat GC somatotropes. (a) Family of representative traces of the inactivation curve in the steady state for the peaks of voltage-dependent *I*_K_ current generated by a double-pulse protocol of −130 mV to +60 mV, with a duration of 1.5 seconds and increases of 10 mV before passing to a testing pulse at 70 mV with a duration of 500 ms; the initial holding potential was 80 mV. (b) The *I*_K_ peak evoked during the testing pulse at +60 mV was normalized in regard to the maximum (*I*/*I*_max_) and graphed in relation to the conditioning pulse voltage.

To determine the proportion of the total K^+^ current in the GC cells evoked at different membrane potentials, the inactivation properties in a steady state of the K^+^ current were examined by using a double-pulse protocol. The protocol consisted of a series of conditioning pulses of varying amplitude, with a duration of 1.5 seconds, ranging from −130 to 60 mV in 10 mV increases and followed by a conditioning pulse at 70 mV with a duration of 500 ms (Figure 3(a)).

A simple Boltzmann relationship could not be adjusted to the data because the presence of rapid and slow inactivation components was indicated. The current at the peak of the testing pulse between the maximum current (*I*/*I*_max_) was normalized and was graphed in relation to the conditioning pulse voltage to obtain the participation percentages of the *I*_A_ and *I*_K_ currents, Figure 3(b) showing that the participation of the *I*_A_ current is below 0.4 (40%). 

### 3.2. Effect of Chronic Treatment with Secretagogues (Ghrelin or GHRP-6) on the Voltage-Dependent Potassium Current in the Cellular Line of Rat GC Somatotropes

**Figure 4:**
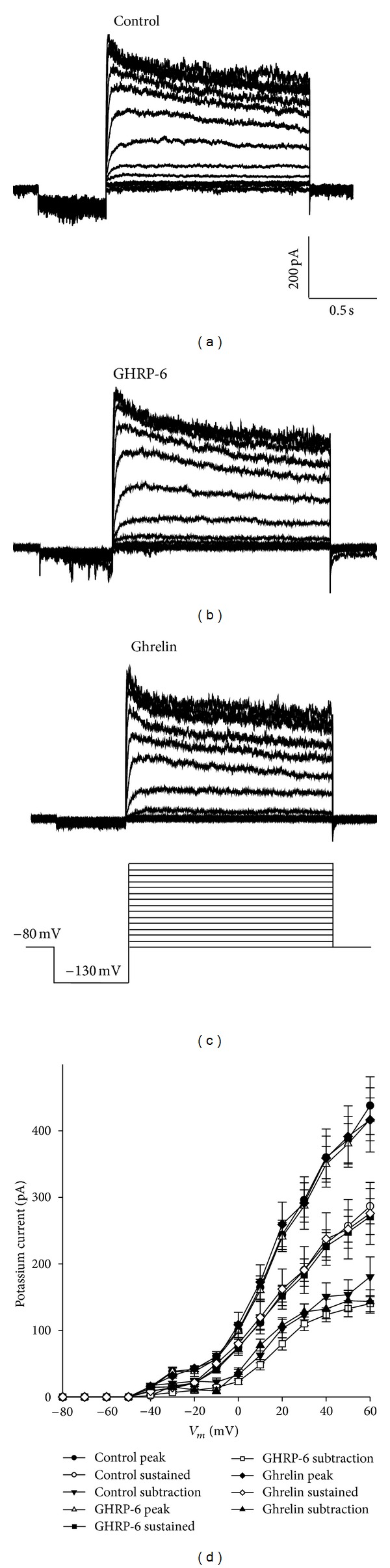
Effect of secretagogues (ghrelin and GHRP-6) on the K^+^ current. (a), (b), and (c): family of representative traces of the K^+^ current on GC cells in control conditions, treated with ghrelin (10 nM), or GHRP-6 (100 nM), for 96 hours. (d) Current-voltage relationship for each one of the experimental conditions (control; *n* = 21, ghrelina 10 nM; *n* = 15, and GHRP-6 100 nM; *n* = 21); the measurements were taken at the peak (the first 50 ms), the sustained component (the last 5 ms; 1.455–1.5 s), and the subtraction of peak minus sustained component.

The chronic effect promoted by ghrelin or GHRP-6 on the voltage-dependent potassium current was evaluated in the GC cells, which were treated for 96 h with ghrelin (10 nM) or GHRP-6 (100 nM).  Figure 4 shows that the chronic treatment with ghrelin or GHRP-6 has no effect on the voltage-dependent potassium current in GC cells. The measurements of the current were performed at the peak (the first 50 ms), the sustained component (the last 5 ms; 1.455–1.5 s), and the subtraction of both measurements.

**Figure 5:**
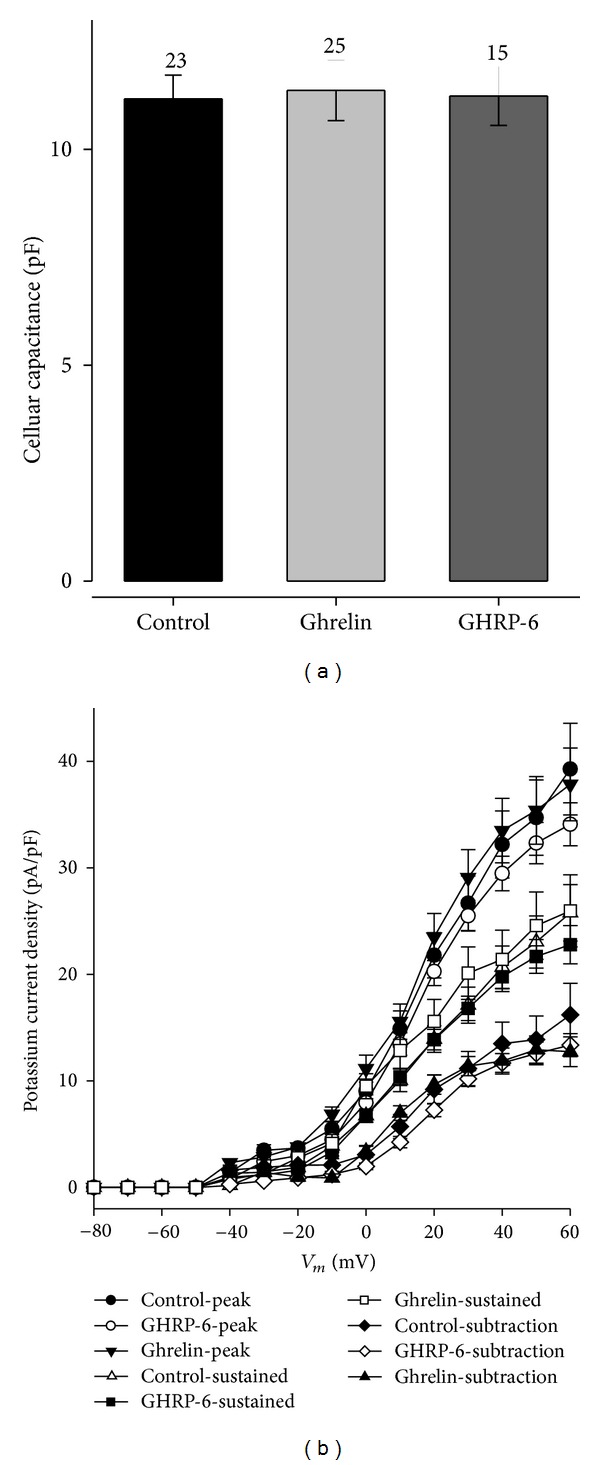
Density of K^+^ current in the cellular line of rat GC somatotropes in control conditions and treated chronically with secretagogues (ghrelin or GHRP-6). (a) Cellular capacitance in each one of the experimental conditions. The numbers beside the error bar represent the number of cells analyzed. (b) Density of current-voltage relationship in the GC cells in control conditions and chronically treated (96 hrs) with ghrelin (10 nM) or GHRP-6 (100 nM).

Together with the results from the voltage-dependent K^+^ current, the density value of the current was obtained in order to eliminate the cell size as a source of variation. The results obtained from this measurement show that the current density is not modified in regard to the control value through chronic treatment with ghrelin or GHRP-6 (Figure 5). 

In order to examine in more detail whether ghrelin or GHRP-6 modified the transient (*I*_A_) or delayed-rectifier (*I*_K_) proportion of the potassium current, specific blockers were employed for each of the currents that comprise the total current recorded. A depolarizing pulse of 60 mV was applied, starting from a prepulse of 500 ms with a membrane potential fixed at −130 mV, the holding membrane potential was −80 mV. 

**Figure 6:**
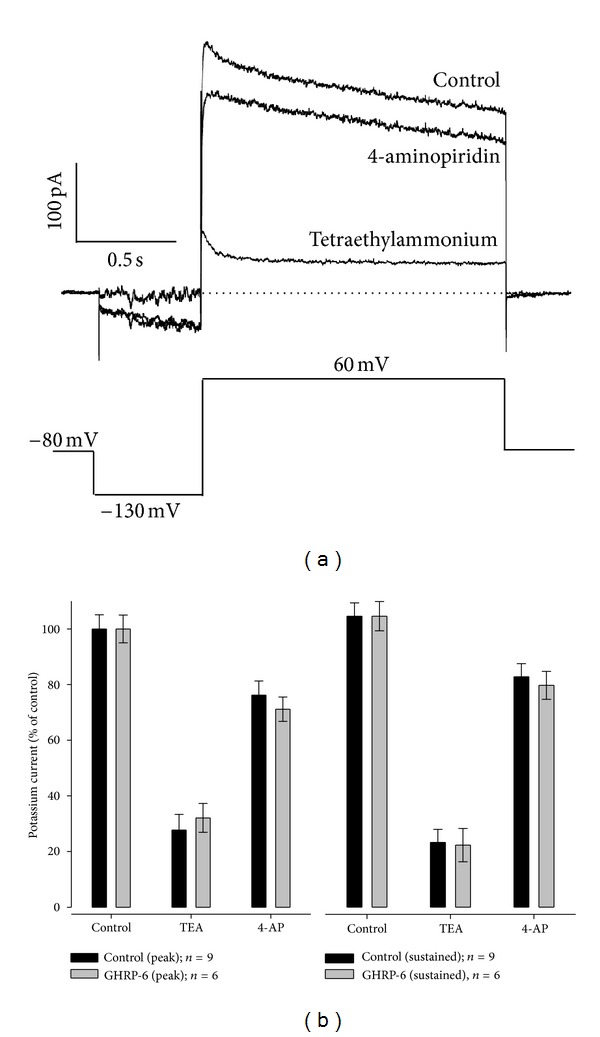
Effect of chronic treatment with secretagogues (ghrelin or GHRP-6) on the percentage of the components of the voltage-dependent potassium current on the cellular line of rat GC somatotropes. (a) Representative traces of a control cell. The current was evoked upon passing from a holding potential from −80 mV to −130 mV for 500 ms, from there to depolarize the cell at 60 mV for 1.5 seconds in order to obtain the totality of K^+^ current. Subsequently, an extracellular solution supplemented with specific blockers for the *I*_K_ (tetraethylammonium; TEA 30 mM) and *I*_A_ currents (4-aminopyridine; 4-AP 5 mM) was perfused with the aim of desiccating the components of the K^+^ current. It should be noted that, before a blocker was used, the control solution was employed for washing the effect of the blocker. (b) Summary of the results obtained by using blockers in the control cells (*n* = 9) and in the cells treated chronically with GHRP-6 100 mM (*n* = 6). The measurements were made at the peak in the first 50 ms, and in the sustained component of the trace in the last 5 ms. The peak and sustained values of the current in the control cells were 314  ±  47 and 200  ±  27 pA, respectively, and for the cells treated chronically with GHRP-6 (100 mM), they were 297 ± 35 and 198  ±  21 pA, respectively.

When the total potassium current had been obtained, it was prefused with an external solution supplemented with 4-aminopyridine at a concentration of 5 mM to block the *I*_A_ current, then an extra cellular solution supplemented with tetraethylammonium (TEA) at a concentration of 30 mM was applied to block the *I*_K_ current. The results are shown in Figure 6.

In Figure 6, it can be observed that the chronic treatment with GHRP-6 does not significantly affect the proportion of the voltage-dependent K^+^ current in its transient (*I*_A_) component, sensitive to 4-aminopyridine, and its sustained (*I*_K_) component, sensitive to TEA.

**Figure 7:**
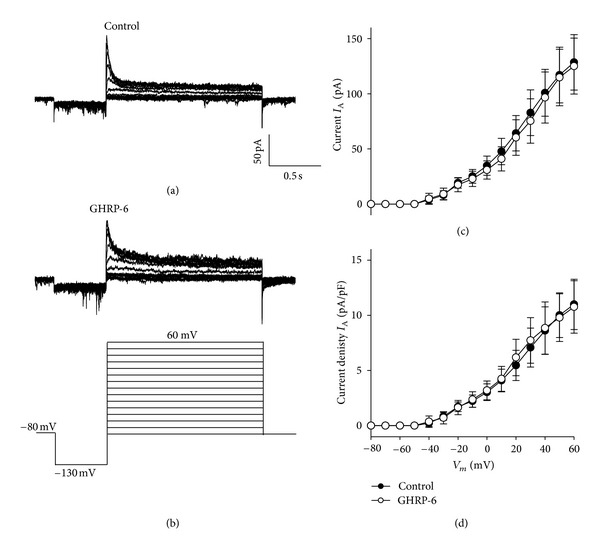
The transient potassium current (*I*_A_) in the cellular line of rat GC somatotropes under control conditions and treated with GHRP-6. (a) and (b): family of representative traces of the *I*_A_ current of a control cell and a cell treated with GHRP-6 (100 nM) for 96 h. The current was evoked by means of a protocol of depolarizing pulses, starting from a holding potential of −80 mV. Thereafter, the cell was hyperpolarized at −130 mV for 500 ms, and then depolarized from −80 to 60 mV with increases of 10 mV lasting 1.5 s; the recording protocol is shown in the lower portion of panel (b). The external recording solution for potassium currents contained Tetraethylammonium 30 mM. (c) and (d): Current-voltage relationship and density of current-voltage, respectively, for each one of the experimental conditions; the peak current was measured in the first 50 ms of the trace. The number of cells analyzed in control condition was 16, with a capacitance value of 11.46 ± 2.86 ee; 14 cells that had been treated with GHRP-6 100 nM for 96 h were also analyzed, their capacitance value being 9.93 ± 2.65 ee.

Subsequently, it was decided to analyze the effect of GHRP-6 on the transient (*I*_A_) current in more detail by using Tetraethylammonium at a concentration of 30 mM in the external recording solution for the purpose of blocking the sustained (*I*_K_) component, which would make it possible to determine whether or not GHRP-6 affects the potassium current in its transient component. The results obtained from these experiments are shown in Figures 7(a) and 7(b).

Chronic treatment (96 h) with the synthetic analogue of ghrelin, GHRP-6 100 nM, does not modify the transient K^+^ current (*I*_A_). The peak value (Figures 7(c) and 7(d)) was obtained by measuring the current during the first 50 ms of the trace. 

**Figure 8:**
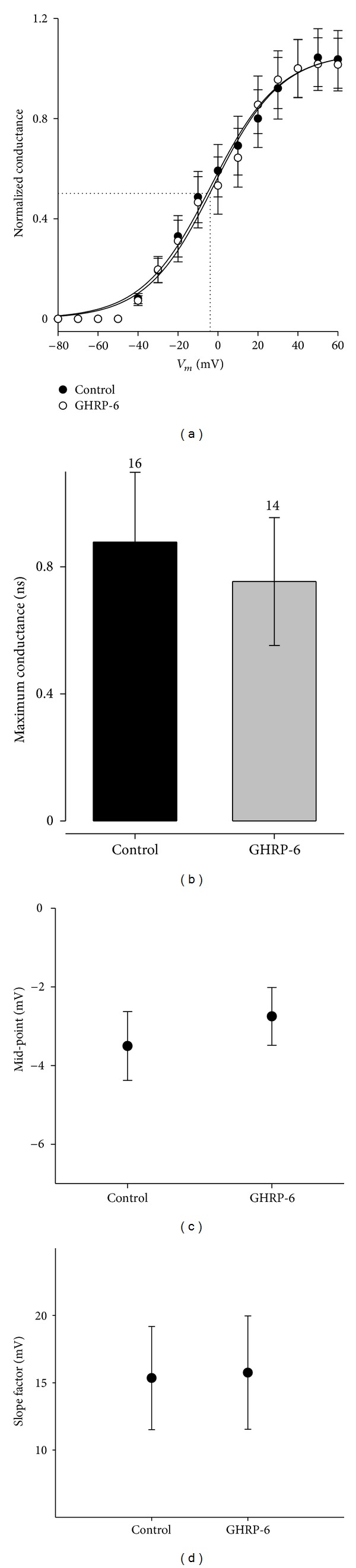
Activation curves of the voltage-dependent potassium current *I*_A_ in the cellular line of rat GC somatotropes. (a) Activation curves of the *I*_A_ current for control conditions and treated with GHRP-6 100 nM for 96 h. The conductances were obtained and these were normalized in regard to the maximum value. The continuous lines represent the adjustment of data with a Boltzmann-type equation. (b) Maximum conductance value for each one of the experimental conditions; the numbers beside the error bar represent the number of cells analyzed. (c) and (d): Boltzmann-type adjustment values, midpoint (*V*_1/2_) and slope factor (*k*), respectively. The adjustments were obtained from the cell-current data shown in Figure 7.

From this same series of experiments, it was possible to obtain the activation curves of *I*_A_ current by adjusting the data to a Boltzmann-type equation (Figure 8). A value of −74.89 mV was obtained for the equilibrium potential of the potassium ion (*E*_K_) with Nerst's equation from our recording solutions. As one can observe, the parameters of the activation curve, *V*_1/2_ (midpoint) and *k* (slope factor), which determine the position and the form of the adjusted curve in the voltage axis, were not modified; likewise, the maximum conductance suffered no significant change (*P* > 0.05) in the chronic treatment with GHRP-6 100 nM.

**Figure 9:**
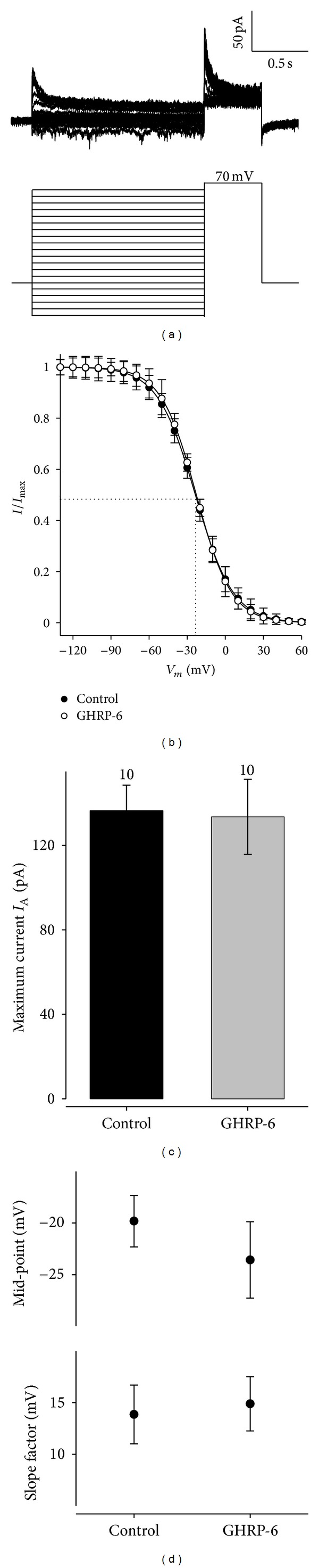
Inactivation in the steady state of *I*_A_ current in the cellular line of rat GC somatotropes. (a) Family of representative traces of the inactivation in the steady state of *I*_A_ current. The current was evoked by a double-pulse protocol; the first of these, named the conditioning pulse, starts from −130 mV and has a duration of 1.5 seconds and a varied amplitude (10 mV); whereas the second one, named testing, carries the membrane potential at 70 mV and lasts 500 ms. The holding potential was fixed at −80 mV. (b) Adjustment of the current at the peak of the testing pulse in accordance with the conditioning pulse voltage and normalization of the latter as regards to the maximum current; the continuous lines represent the adjustment of the data with a Boltzmann-type function. (c) Maximum current evoked by the testing pulse in experimental conditions; the numbers beside the error bar represent the number of cells analyzed. (d) Boltzmann-type adjustment of values: midpoint (*V*_1/2_) and slope factor (*k*).

In the same way, the inactivation of the *I*_A_ current in control conditions and treated with GHRP-6 100 nM for 96 h was evaluated in order to discard any change in the kinetics of the potassium channels in charge of type *I*_A_ current (Figure 9). A double-pulse protocol was used to construct the inactivation curve, the first pulse being designated as conditioning and the second one as testing (see data analysis). The peak amplitude of the *I*_A_-type current induced by the testing pulse was graphed according to the voltage of the conditioning pulse and the data obtained from each cell were adjusted with a Boltzmann-type equation; then the data and the adjusted function were normalized in regard to the *I*_max_ value. The result of the inactivation curves with the *I*_A_ current in a steady state is shown in Figure 9. Chronic treatment with GHRP-6 100 nM does not modify the kinetic macroscopic properties of type *I*_A_ K^+^ current, since the parameters of *V*_1/2_ and of *k* are not affected (Figures 9(c) and 9(d)).

### 3.3. Chronic Effect of Secretagogues (Ghrelin or GHRP-6) on the Delayed-Rectifier K^+^ Current (*I*_K_) in GC Cells

**Figure 10:**
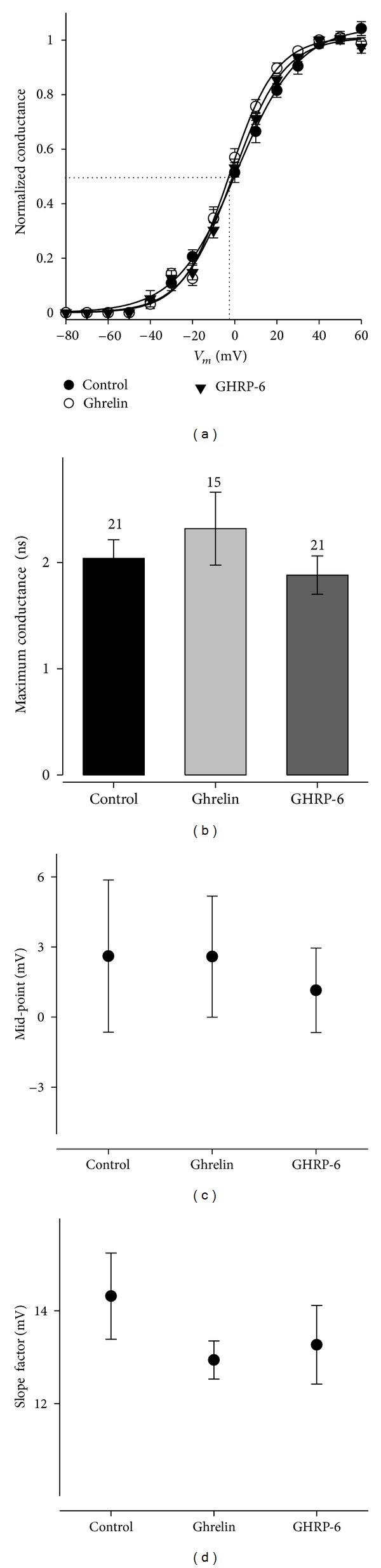
Activation curve of the sustained component of the voltage-dependent K^+^ current in the cellular line of rat GC somatotropes. (a) Adjustments to the normalized conductance of the GC cells under control conditions, treated with ghrelin 10 nM and GHRP-6 100 nM for 96 h. The data were obtained from the current-voltage curves in Figure 4, the continuous lines representing the adjustment of data with a Boltzmann-type function. (b) Maximum conductance value for each of the experimental conditions. (c) and (d): values of the Boltzmann-type adjustment parameters for each of the experimental conditions, midpoint (*V*_1/2_) and slope factor (*k*), respectively.

We evaluated the chronic effect exercised by ghrelin and by its synthetic analogue GHRP-6 on the sustained component of the voltage-dependent potassium current named delayed rectifier (*I*_K_). The values obtained from the current-voltage curves shown in Figure 4 were used to calculate the activation curve. The data obtained were normalized in regard to the maximum conductance value and were later adjusted to a Boltzmann-type function, the results of which are shown in Figure 10.

Figure 10 shows the results of the activation curves of the *I*_K_ current. The chronic treatment with ghrelin or GHRP-6 does not modify the kinetic properties of *I*_K_ current, since the voltage value at which 50% of the channels are activated is not significantly different (*P* > 0.05), the same as the value of *k*, which determines the form of the activation curve.

**Figure 11:**
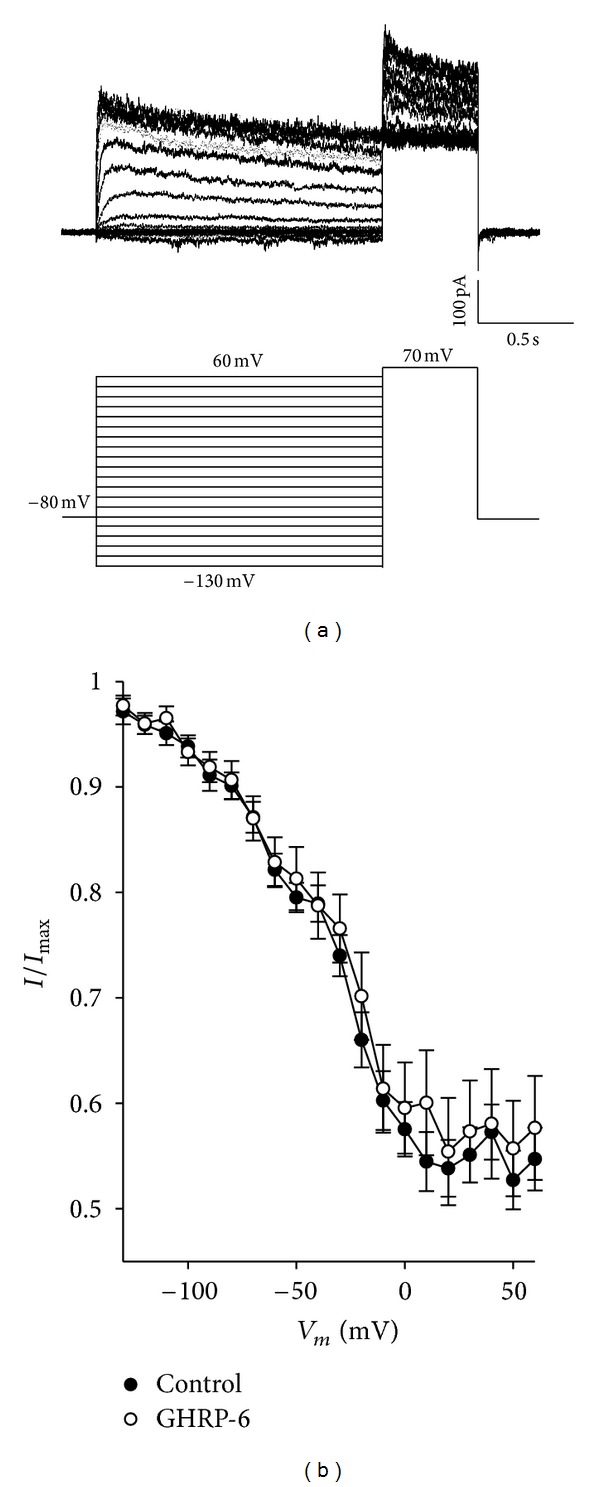
Inactivation in steady state of the total voltage-dependent K^+^ current in the cellular line of rat GC somatotropes. (a) Family of representative traces of the steady-state inactivation for the total voltage-dependent K^+^ current. The current was evoked by a double-pulse protocol. The first pulse, named conditioning, starts from a membrane potential of −130 mV, having duration of 1.5 sec and varying amplitude; the second one, testing, carries a membrane potential of 70 mV with duration of 500 ms. (b) The current at the peak of the testing pulse was graphed according to the voltage of the conditioning pulse, this being normalized in regard to the maximum current.

Otherwise, it was decided to evaluate the kinetics of inactivation in a steady state of the total potassium current under control conditions and treated with GHRP-6 (100 nM) for 96 h. A double-pulse protocol was used for this purpose, and the current was evoked by a double-pulse protocol (see data analysis) (Figure 11(a)). The current was graphed at the peak of the testing pulse in accordance with the voltage of the conditioning pulse, this being normalized in regard to the maximum current (Figure 11(b)).

As one can observe in Figure 11, chronic treatment with GHRP-6 100 nM does not modify the inactivation kinetics of the total K^+^ current, the conclusion being that neither ghrelin nor GHRP-6 modifies the potassium current.

### 3.4. The Effect of Chronic Treatment with GHRP-6 on the Potassium-Inward-Rectifier (KIR) Current in the Cellular Line of Rat GC Somatotropes

**Figure 12:**
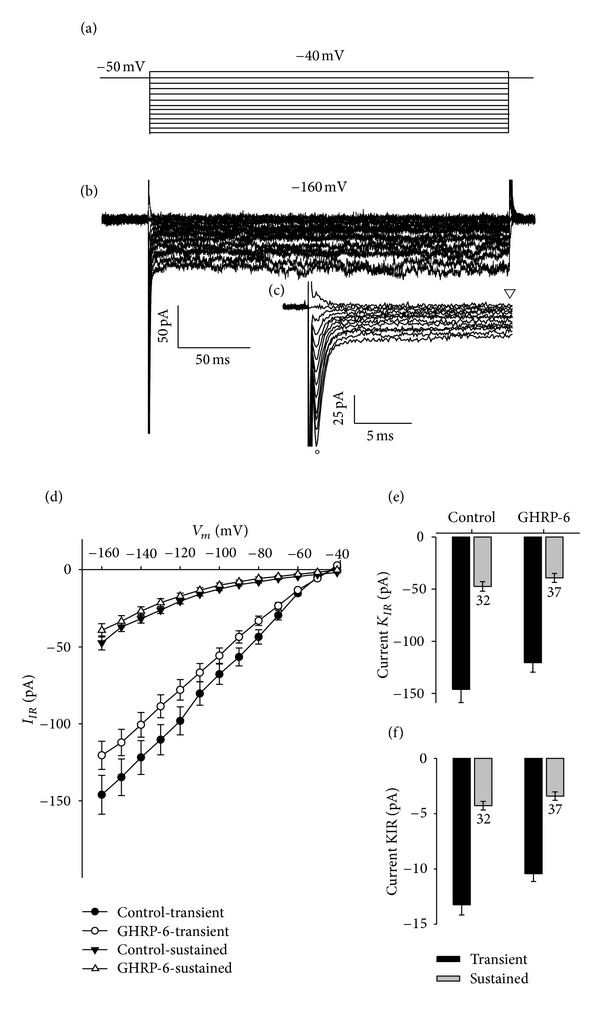
Effect of GHRP-6 on the K^+^ inward-rectifier current (KIR) in the cellular line of rat GC somatotropes. (a) Recording protocol to evoke the inward-rectifier current in GC cells, taken from Xu et al. [15], The protocol consists of hyperpolarizing pulses from −160 to −40, starting from a holding potential of −50 mV and a duration of 250 ms in steps of 10 mV, with the same recording solution for the K^+^ *I*_K_ current. (b) Family of KIR current traces showing two components, an initial (transient) one of rapid activation and inactivation in the first 2-3 ms, followed by a (sustained) component of slow activation that remains for the 250 ms duration of the pulse. The transient component was measured 1 ms after the end of the capacitive component to avoid contamination from the component, the latter having a duration of ~0.3 ms; the sustained component was measured 5 ms before the end of the current trace (245–250 ms). (c) Insert of the same trace shown in (b), which was expanded in time so as to observe the transient component of the KIR current. (d) Current-voltage curves of the two KIR components. (e) and (f): current and density of current measured in the trace at −160 mV. The capacitance value for the control cells was 11.02 ± 0.50 and for the cells treated with GHRP-6 it was 11.50 ± 0.58; the numbers beside the error bars show the number of cells analyzed.

Figure 12(a) shows the recording protocol [15] for evoking the rectifying current entering the GC cells. The protocol consists of hyperpolarizing pulses from −160 to −40, starting from a holding potential of −50 m and a duration of 250 ms in steps of 10 mV, with the same recording solution used for the K^+^ current (*I*_K_) Figure 12(b) shows a family of currents presenting two components of the KIR current, an initial (transient) component of rapid activation and inactivation in the first 2-3 ms, followed by a (sustained) component of slow activation that remains during the 250 ms of the pulse. The transient component was measured 1 ms (empty and full circles) after the end of the capacitive component in order to avoid contamination from the latter, which had a duration of ~0.3 ms; the sustained component was measured 5 ms (empty and full inverted triangles) before the end of the current trace (242–250 ms). Figure 12(c) shows an insert of the same trace shown in part Figure 12(b), which was expanded in time in order to observe the transient component in more detail. The current-voltage curves of the two KIR components exhibit kinetics of inward rectification, especially for the sustained part of the KIR current (Figure 12(d)). 

The chronic treatment with GHRP-6 (100 nM) does not significantly modify (*P* < 0.05) the current or the density of the potassium KIR current in its two components, transient and sustained, measured in the step of voltage at −160 mV (Figures 12(e) and 12(f)).

**Figure 13:**
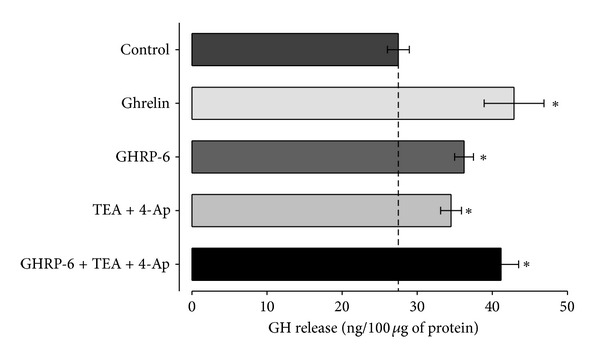
The treatment with secretagogues (ghrelin or GHRP-6) does not affect the functional expression of voltage-dependent K^+^ channels in the cellular line of rat GC somatotropes. The bars in the graph illustrate the regulation of GH liberation by ghrelin (10 nM) or GHRP-6 (100 nM), applied alone or in the presence of a selective blocker of potassium channels (TEA; 30 mM and 4 Ap; 5 mM). Each value represents the mean ± EE of the determinations carried out in triplicate of three independent experiments. The asterisks denote significant differences (*P* < 0.05) compared with the untreated (control) cells.

Finally, in order to investigate the possible contribution of the voltage-dependent potassium channels to the secretion mediated by the GH secretagogues in the GC cells, ELISA experiments using drugs affecting the activity of the channels were carried out. To investigate the participation of the potassium channels, the GC cells were incubated for 96 hours under control conditions or treated by ghrelin (10 nM) or GHRP-6 (100 nM), which tend to increase the secretion of GH (~35%) with regard to the control value (Figure 13), were used alone and in combination with a selective blocker of potassium channels (TEA, 30 mM, and 4 Ap, 5 mM). The secretion of GH in cells treated with GHRP-6 combined with the potassium blockers was not different from the secretion provoked by GHRP-6 or ghrelin alone. Upon analyzing the GH secretion in cells treated with TEA and 4 Ap, an increase is observed in regard to control value which, however, does not differ from that occasioned by GH secretagogues. Therefore, we may conclude that the secretagogues do not affect the functional expression of the voltage-dependent potassium channels. 

## 4. Discussion

GH is an anabolic hormone that regulates both growth and development in many species [24]. It is well established that the secretion of GH occurs under the neuroendocrine control of GHRH and of somatostatin at the pituitary level, with an additional regulation proportioned by ghrelin [2, 11, 12]. Ghrelin, an endogenous ligand for GHS-R, is apparently involved in an additional neuroendocrine pathway to control the secretion of GH [12, 14]. For this reason, ghrelin strongly stimulates the liberation of GH, in vitro and in vivo, in a wide range of species, including humans [2, 11, 12, 14]. 

The present study was undertaken to examine the effect of the long-term incubation with secretagogues of GH (ghrelin or GHRP-6) on the functional expression of potassium channels in the cellular line of rat GC somatotropes, which is a subclone of GH_3_ cell line. The results indicate that the voltage-dependent K^+^ currents (*I*_A_, *I*_K_ and KIR) do not suffer any significant changes, neither in their kinetic properties of activation nor in their inactivation through treatment with secretagogues. Previously, Chen [11] demonstrated that the K^+^ current increases through long-term treatments (48 hrs) with GHRP-2 by means of an increase in protein synthesis for the K^+^ channel in ovine somatotropes, this effect is mediated by the pathway PKC system.

The effect of ghrelin on GH secretion is thought to be connected with various systems of signaling. In pigs, the liberation of GH in response to ghrelin depends on signaling systems that involve cAMP/PKA as well as PLC/PKC pathways, and on the influx of extracellular Ca^2+^ [4]. In rats, the liberation of ghrelin-dependent GH is provoked by both intracellular Ca^2+^ liberation and extracellular Ca^2+^ influx [25]; the influx of Ca^2+^ is via voltage-dependent Ca^2+^ channels that are activated through depolarization [25, 26]. In tumorous GC cells, it has been reported that long-term exposure (96 h) with ghrelin stimulates the functional expression of the voltage-dependent Na^+^ and Ca^2+^ channels involved in the influx of extracellular calcium to promote the secretion of GH [26, 27]; in contrast, other researchers have reported that ghrelin reduces the voltage-dependent Ca^2+^ current via GMP in GH_3_ cells [28].

The electrical activity of the somatotropes depends on the properties as well as on the functional expression of the ionic channels present in the plasmatic membrane and on the potential of the membrane in repose. The K^+^ current flowing through the plasmatic membrane is responsible for the potential of the membrane in repose, although other ions such as Na^+^ and Ca^2+^ may be involved [17]. Based on their properties through differences in time, voltage dependency, and pharmacological sensitivity, various types of K^+^ current have been identified in the somatotropes [29, 30]; this currents include inward rectifiers (KIR), transient current (*I*_A_), delayed rectifier current (*I*_K_), sometimes called IRD, K^+^ currents activated by [Ca^2+^]_*i*_ [30, 31]. Both currents, *I*_A_ and *I*_K_, are involved in the electrical activity of somatotropes [29–31]. The *I*_A_ is partly responsible for maintaining the membrane potential in repose and participating in the repolarization process of the potential in action [29–32]. The role of the *I*_K_ current carried by voltage-dependent K^+^ channels in electrical activity has been examined in various cellular types; in GH_3_ cells, the inhibition of this channel by TEA has been seen to increment the duration of the action potential [33], as well as the amplitude of the spontaneous transients of [Ca^2+^]_*i*_ [34]; in rat lactotropes TEA does not modify the firing pattern [33]. Somatostatin increases both the *I*_K_ and the KIR in rat, ovine and human somatotropes [18, 19, 35, 36].

On the other hand, GHRH reduces the K^+^ current in human adenoma cells as well as in GH_4_C_1_ cells [37, 38]; the synthetic analogue of ghrelin, GHRP-6, diminishes both the transient and the delayed rectifier currents in rat somatotropes [39]. However, an increase in the voltage-dependent K^+^ current (both *I*_K_ and *I*_A_) has been reported, occasioned by an increase in the synthesis of protein that codifies for the K^+^ channel provoked by another GHS, GHRP-2 [40]. Up to now, there are few reports regarding the effect of ghrelin or GH secretagogues on the functional expression of ionic channels and the routes they may be occupying [26, 27, 40, 41]. It has been reported that ghrelin inhibits the inward rectifier of the K^+^ channel coupled with protein G in neurons of the tuberous mammillary nucleus (KIR_3_) [42]. In the present work, we demonstrate that ghrelin does not affect the K^+^ currents (*I*_K_ and *I*_A_) during a long-term exposure (96 h) in the cellular line of rat GC somatotropes. In work realized on GH_3_ cells, ghrelin acutely (in the bath) reduces the voltage-dependent K^+^ current; this effect of ghrelin, mediated by means of the cGMP/PKG system [20], occurs through the activation of a GHS receptor, since GHRH-R is not present in these cells [43].

In primary cultures of pituitary cells and in GH_3_ cells, it has been shown that the inhibition of the KIRs can generate a higher firing rate of action potentials and subsequently an increase in the secretion of the prolactin hormone and GH [44]. Treatment with ghrelin or GHRP-6 does not significantly modify the KIR current in these cells at physiological voltages; however, a reduction of KIR current has been reported in ovine somatotropic cells with GHRP-2 through the PKA-AMPc pathway [15]. To summarize, chronic treatment with ghrelin or GHRP-6 does not modify the functional expression of the K^+^ channels underlying the *I*_K_, *I*_A_, and KIR currents in the cellular line of rat GC somatotropes, subclone of GH_3_.
